# Guanidino-Acetic Acid: A Scarce Substance in Biomass That Can Regulate Postmortem Meat Glycolysis of Broilers Subjected to Pre-slaughter Transportation

**DOI:** 10.3389/fbioe.2020.631194

**Published:** 2021-02-10

**Authors:** Bolin Zhang, Ning Liu, Zhen He, Peiyong Song, Meilin Hao, Yuxiao Xie, Jiahui Li, Rujie Liu, Zewei Sun

**Affiliations:** ^1^Department of Biology and Agriculture, Characteristic Laboratory of Animal Resources Conservation and Utilization of Chishui River Basin, Zunyi Normal College, Zunyi, China; ^2^College of Animal Science and Technology, Jilin Agricultural University, Changchun, China

**Keywords:** guandino-acetic acid, transport stress, postmortem glycolysis, AMPK signaling, broiler

## Abstract

The different substances in biomass can regulate the metabolism and reproduction of broilers. Guanidino-acetic acid (GAA) is a natural feed additive that showed a potential application in dietary for broilers, while its amount is scarce in biomass. The objective of the present study was to investigate the effects of dietary supplemented with GAA on muscle glycolysis of broilers subjected to pre-slaughter transportation. A total of 160 Qiandongnan Xiaoxiang chickens were randomly assigned into three treatments, including a basal control diet without GAA supplementation (80 birds) or supplemented with 600 mg/kg (40 birds) or 1,200 mg/kg (40 birds) GAA for 14 days. At the end of the experiment, the control group was equally divided into two groups, thus resulting in four groups. All birds in the four groups aforementioned were separately treated according to the following protocols: (1) no transport of birds of the control group fed with the basal diet; (2) a 3-h transport of birds of the control group fed with the basal diet; (3) a 3-h transport of birds fed with diets supplemented with 600 mg/kg GAA; and (4) a 3-h transport of birds fed with diets supplemented with 1,200 mg/kg GAA. The results demonstrated that 3-h pre-slaughter transport stress increased corticosterone contents and lowered glucose contents in plasma (*P* < 0.05), decreased pH_24__h_ (*P* < 0.05), and resulted in inferior meat quality evidenced by elevating the drip loss, cooking loss, and L^∗^ value (*P* < 0.05). Meanwhile, 3-h pre-slaughter transport stress decreased the contents of Cr and ATP in muscle (*P* < 0.05) and elevated the ratio of AMP:ATP and the glycolytic potential of muscle (*P* < 0.05). Moreover, 3-h pre-slaughter transport resulted in a significant elevation of mRNA expressions of LKB1 and AMPKα2 (*P* < 0.05), as well as the increase in protein abundances of LKB1 phosphorylation and AMPKα phosphorylation (*P* < 0.05). However, 1,200 mg/kg GAA supplementation alleviated negative parameters in plasma, improved meat quality, and ameliorated postmortem glycolysis and energy metabolism through regulating the creatine–phosphocreatine cycle and key factors of AMPK signaling. In conclusion, dietary supplementation with 1,200 mg/kg GAA contributed to improving meat quality via ameliorating muscle energy expenditure and delaying anaerobic glycolysis of broilers subjected to the 3-h pre-slaughter transport.

## Introduction

Pale, soft, and exudative (PSE)-like meat, characterized by lighter appearance, softer texture, lower water holding capacity, excessive yield losses, and formation of soft gels, has been recognized for decades and causes huge economic losses annually for modern poultry industry due to its inferior quality (Desai et al., 2016; Wang et al., 2017). In previous studies, it has been suggested that a 3-h pre-slaughter transport resulted in a significant decrease of glucose concentration in plasma and a sharp increase in plasma corticosterone level (Zhang et al., 2014, 2017), which are the two most sensitive indicators of transport stress. Moreover, pre-slaughter transportation accelerated energy consumption in muscle of broilers and resulted in a stress-induced increase in glycolysis, further inducing the accumulation of lactic acid in muscle (Hambrecht et al., 2005; Zhang et al., 2009). It has been well established that fast and excessive glycolysis, a rapid accumulation of lactic acid, and high temperature in muscle of early postmortem are the causes of PSE-like syndrome (Li et al., 2016). Therefore, pre-slaughter transport was considered to be one of the most important factors contributing to an increase in incidences of PSE-like chicken meat (Huang et al., 2018). Moreover, Xing et al. (2016) demonstrated that pre-slaughter transport induced a lower energy status in the early postmortem period, followed by an elevation of the concentration of lactic acid and an inferior meat quality. As a result, pre-slaughter transportation is one of the most important pre-harvest variables related to meat quality and should be regarded as a critical control point (Speer et al., 2001; Schwartzkopf-Genswein et al., 2012). Therefore, it is necessary to take a nutritional strategy to improve energy storage and alleviate the adverse effects of pre-slaughter transportation on meat quality of broilers.

Creatine (Cr) is heavily involved in energy metabolism through the Cr and phosphocreatine system, an important cellular energy source for rapid regeneration of ATP to meet the increased energy demands of intense activities of tissues, particularly muscle cells (Michiels et al., 2012; Zhang et al., 2017). It has been demonstrated that Cr, a natural regulator of energy homeostasis, could buffer energy concentration in tissues with significant and fluctuating energy demands, especially in muscles and brains (Ostojic et al., 2013). Guanidino-acetic acid (GAA) is the only immediate precursor of Cr in the body and is a naturally occurring compound in vertebrate animals (Dilger et al., 2013). Importantly, GAA is more chemically stable and more effective than Cr at enhancing tissue Cr storage (McBreairty et al., 2015). Accordingly, GAA was considered to be more suitable than Cr as a new natural feed additive. It has been demonstrated that dietary supplemented with GAA increased the contents of Cr and phosphorus creatine (PCr) in muscle and improved the state of energy metabolism (Degroot, 2015). A previous study demonstrated that the increased concentrations of Cr and PCr and the higher ratio of PCr: Cr were observed by GAA supplemented in the diet (Liu et al., 2015). In addition to being involved in the regulation of energy metabolism, GAA also played an important role in improving meat quality. It has been reported that GAA supplementation increased postmortem muscle pH, lowered drip loss and cooking loss, and significantly reduced the shear force of meat (Wang et al., 2012). This may be due to the fact that dietary GAA supplementation increased available energy reserve such as PCr and ATP, which contributes to delaying the energy release of glycolysis and the accumulation of lactic acid, and consequently maintained a pH value postmortem. Moreover, Liu et al. (2015) suggested that GAA supplementation significantly decreased the activity of hexokinase, the rate-limiting enzyme in glycolysis, which means that GAA regulated the process of muscle energy metabolism through postmortem muscle glycolysis. However, the regulation mechanisms of GAA on postmortem glycolysis are still unknown.

Adenosine 5′-monophosphate (AMP)-activated protein kinase (AMPK), the downstream component of the protein kinase signal cascade pathway, is a key factor in sensing intracellular energy status and acts as a crucial component in regulating energy balance at both the cellular and whole body levels (Hardie, 2011). AMPK is an important energy sensor in the body, which is called the “energy regulator” of eukaryotic cells. AMPK was activated in response to stresses such as the exhaustion of ATP and the increase of the proportion of ADP/ATP, consequently accelerating postmortem glycolysis (Graeme and Hardie, 2014). Besides, postmortem glycolysis was inhibited in AMPK knockout mice, suggesting that APMK could be a target to control postmortem glycolysis (Shen et al., 2008). Thus, we hypothesized that GAA regulated the postmortem glycolysis through the AMPK signaling pathway. Therefore, the objective of our study was to evaluate the effects of GAA supplementation on meat quality and intracellular energy metabolism of muscle, further determining the activities of key enzymes involved in glycolysis and gene or protein expressions of important components in the AMPK signaling pathway of broilers subjected to pre-slaughter transport.

## Materials and Methods

### Animal Care, Diet, and Management

All the procedures including animal care and experiment treatments were in accordance with guidelines approved by the Institutional Animal Care and Use Committee of Zunyi Normal College. The basal diet was formulated to meet the nutrient requirements of Nutrient Requirements of Chinese Meat-Type Yellow Feathered Chickens. The composition and nutritional level of the diet are as shown in Table 1.

**TABLE 1:** Composition and nutrient levels of the basal diet (air-dry basis,%).

**Items**	**Contents**	**Nutrient levels**	**Contents**
Corn	68.50	^2^Crude protein (%)	16.00
Soybean meal	12.00	Metabolic energy (MJ/kg)	13.03
Corn gluten meal	3.60	Lysine (%)	0.85
Rapeseed meal	1.95	Methionine (%)	0.65
Soybean oil	3.93	Calcuim (%)	0.81
L-Lysine HCl	0.32	Available phosphorus (%)	0.35
DL-Methionine	0.10		
Calcuim monophosphate	1.30		
Limestone	0.85		
Salt	0.30		
1% Premix^1^	1.00		

*^1^Premix provided the following per kg of the diet: vitamin A 10,000 IU; vitamin D3 2,000 IU; vitamin E 25 mg; Vitamin K, 2.8 mg; thiamine 2.50 mg; riboflavin 7.5 mg; nicotinamide 40 mg; calcium pantothenate, 25 mg; pyidoxine⋅HCl, 3 mg; biotin, 0.20 mg; folic acid, 1.5 mg; vitamin B12, 0.015 mg; ferrous (as ferrous sulfate) 80 mg; copper (as copper sulfate) 8 mg; manganese (as manganese sulfate) 100 mg; zinc (as zinc sulfate) 60 mg; iodine (as potassium iodide) 0.35 mg; selenium (as sodium selenite) 0.3 mg.*

*^2^Nutrient content of the diets were the value of measurement.*

One hundred and sixty Qiandongnan Xiaoxiang chickens (purchased from Guizhou Rongjiang Shannong Development Co., Ltd., China) 22 weeks old were randomly assigned into three treatments, including a basal control diet without GAA supplementation (80 birds) or supplemented with 600 mg/kg (40 birds) or 1,200 mg/kg (40 birds) GAA for 14 days. The source of GAA additive (>99% purity) was from Tianjin Tiancheng Pharmaceutical Co., Ltd. (Tianjin, China). Each treatment was consisted of 20 replicates and two broilers per cage, but the control group had 40 replicates and two broilers per cage. All birds were housed in an environmentally controlled facility and were *ad libitum* access to the feed in mash form and fresh water. At the end of the experiment, the body weight of the bird as the unit of pen was weighted after an 8-h feed deprivation. Average daily weight gain (ADG) and feed intake (ADFI) were calculated.

### Transportation and Sample Collection

Before transportation treatment, all birds were tagged and fasted overnight without water withdrawal. The control group was averagely divided into two groups, thus resulting in four groups consisting of two control groups and two GAA supplementation groups. All birds in the four groups mentioned above were transported from the rearing house to the slaughterhouse according to the following protocols: (1) no transport of birds of the control group fed with the basal diet; (2) a 3-h transport of birds of the control group fed with the basal diet; (3) a 3-h transport of birds fed with diets supplemented with 600 mg/kg GAA; and (4) a 3-h transport of birds fed with diets supplemented with 1,200 mg/kg GAA.

No water or feed was provided during the transportation period. The transportation distance is about 240 km with an average speed of 80 km/h. After a 3-h transportation and rest for 1 h, 10 broilers with a body weight close to the mean body weight in each group were randomly selected for blood sampling. Blood samples were collected into 10-mL Eppendorf tubes coated with EDTA via wing vein puncture to collect plasma for subsequent analysis.

The left pectoralis major (PM) was taken from broilers within 10 min after slaughter and stored at 4°C for meat quality analysis. In addition, about 5 g of right PM was collected into the frozen tube and stored in liquid nitrogen for further analysis.

### Growth Performance

At the beginning and end of the experiment, the body weights of broilers were separately recorded per pen. Feed consumption was recorded daily. Thus, the ADG, ADFI, and ratio of feed intake to the average daily weight gain (F:G) were calculated for each replicate pen of chicken.

### Concentrations of Glucose and Corticosterone in Plasma

The concentration of glucose was analyzed using commercial test kits (Nanjing Jiancheng Bioengineering Institute, Nanjing, China) according to the protocols of manufacture. The concentration of corticosterone was determined with commercial kits (Cusbio Biotech. Co., Ltd., Wuhan, China).

### Meat Quality

Postmortem pH at 45 min (pH_45__min_) of the left PM were measured with PHBF-260 portable pH meter (Shanghai Instrument Electric Science Instrument Co., Ltd., Shanghai, China) and then were stored at 4°C to determine postmortem pH at 24 h (pH_24__h_). Each sample was measured at 3 locations. The probe of the pH meter was inserted into PM at an angle of 45° and was washed using ultrapure water between different samples. The values of meat color including a^∗^ (redness), b^∗^ (yellowness), and L^∗^ (lightness) at postmortem 24 h of the right PM were determined using a YS3010 portable spectrophotometer (Shenzhen San’enshi Technology Co., Ltd., Shenzhen, China). Drip loss, cooking loss, and shear force of PM were measured according to the protocols reported by the previous studies (Yue et al., 2010; Zhang et al., 2019).

### Concentration of Cr, PCr, and Adenosine Nucleotides in Muscle

The concentrations of Cr, PCr, ATP, ADP, and AMP in muscle were determined by HPLC as previously reported with moderately modified (Wang et al., 2017; Zhang et al., 2017). Three hundred milligram frozen muscle samples were collected into tubes containing ice-cold perchloric acid and were homogenized for 1 min, followed by standing in an ice bath for 15 min. Then, the homogenates were centrifuged at 15,000 *g* at 4°C for 10 min to collect supernatants and further filtered through a 0.45-μm membrane. A 10-μL volume of each sample was injected into an Alliance HPLC system (Alliance HPLC system 2695, Water Corporation, Milford, MA, United States) equipped with a Waters SunFire C18 column (250 mm × 4.6 mm, 5 μm) at a temperature of 25°C for Cr and PCr determination, and at a temperature of 30°C for ATP, ADP, and AMP analysis. The ultraviolet wavelength for Cr and PCr determination and the analysis of ATP, ADP, and AMP were 210 and 245 nm, respectively. The mobile phase was a mixture of methyl cyanides and 29.4 mM KH_2_PO_4_ buffer (2:98, volume ratio) for Cr and PCr determination and was a mixture of methanol and phosphate buffer (13.5:86.5, volume ratio) for ATP, ADP, and AMP analysis, and the flow rate was 1 mL/min. The standards of creatine and phosphocreatine disodium salt, and 5′-ATP disodium salt, 5′-ADP sodium salt, and 5′-AMP sodium salt were all purchased from Sigma-Aldrich, Inc. (Sigma-Aldrich Inc., St. Louis, MO, United States).

### Analysis of Activities of Glycolytic Key Enzymes

The activities of glycolytic key enzymes, including hexokinase (HK), pyruvate kinase (PK), and phosphofructokinase (PFK), were conducted with commercial kits (Nanjing Jiancheng Bioengineering Institute, Nanjing, China). All operation protocols were according to instructions of manufacture.

### Determination of Glycogen, Lactic Acid, and Glycolytic Potential in Muscle

About 0.50 g (weighed exactly) frozen muscle sample was homogenized for 1 min in 4.5 mL ice-cold saline then was centrifuged for 10 min at 4,000 rpm at 4°C to collect superannuants for determining the concentration of lactic acid in muscle with a standard commercial kit (Nanjing Jiancheng Bioengineering Institute, Nanjing, China). Glycogen content was measured as previously reported (Zhang et al., 2009). Both lactic acid and glycogen contents were used to calculate the glycolytic potential (GP) according to the formula: GP = 2 × [glycogen] + [lactic acid], which was expressed as μmol of lactic acid equivalent per gram of wet muscle (Monin and Sellier, 1985).

### RNA Extraction and Real-Time PCR Analysis

The mRNA expressions of the selected genes were evaluated by real-time PCR. Total RNA from muscle samples were extracted using RNAiso Plus reagent (catalog no. 9108, TaKaRa Biotechnology (Dalian) Co., Ltd., Dalian, China) according to the manufacturer’s protocols. The purity of the total RNA was quantified by evaluating the OD260/OD280 ratio with a ND-1000 spectrophotometer (NanoDrop, Thermo Fisher Scientific). Samples with 260/280 ratios of 1.8-\to 2.0 and 260/230 ratios of 2.0 to 2.2 were used for PCR reactions. Reverse transcription was conducted with the PrimeScript^TM^ RT Master Mix (catalog no. RR037A, TaKaRa), and real-time RT-PCR was conducted using TB Green Premix Ex Taq (catalog no. RR420A). The PCR program was as follows: one cycle at 95°C for 30 s and 40 cycles at 95°C for 5 s, followed by 60°C for 30 s. The expression of target genes relative to the housekeeping gene (β-actin) was analyzed according to the method by Livak and Schmittgen (2000). All samples were processed in triplicate. The relative mRNA expression of each target gene was normalized to the control group (no transportation stress). The primer sequences for the target and housekeeping genes are shown in Table 2.

**TABLE 2:** Sequences used for real-time PCR primers.

**Genes**	**Primers (5′→3′)**	**Product size (bp)**	**Gene Bank^1^**
*LKB1*	Sense: GTATGACGGCGGTGCCTTATCTG	121	NM_001045833.1
	Antisense: ACCTGTCCTGGTACTGTGAAGTCC		
*AMPK*α*1*	Sense: TGTGTATGTGCAGCAACCCG	195	NM_001039603.1
	Antisense: AACAACCAGCTATGCACCCC		
*AMPK*α*2*	Sense: TCATCAGCACGCCAACAGACTTC	106	NM_001039605.1
	Antisense: CGAGCCTCTGCCTCTTCAACAC		
β*-actin*	Sense: ATTGTCCACCGCAAATGCTTC	113	NM_205518.1
	Antisense:AAATAAAGCCATGCCAATCTCGTC		

*LKB1, liver kinase B1; AMPKα1, adenosine 5′-monophosphate-activated protein kinase α1; AMPKα2, adenosine 5′-monophosphate-activated protein kinase α2. ^1^Genbank Accession Number.*

### Western Blot

Approximately 10 mg frozen muscle samples was homogenized in tubes with 200 μL lysis buffer, then was centrifuged for 10 min at 12,000 rpm and 4°C to collect the supernatant, followed by protein quantification assay. Each muscle supernatant was mixed with an equal amount of 2× standard SDS sample loading buffer containing 0.5 M Tris–HCl (pH 6.8), 4.4% (w/v) SDS, 20% (v/v) glycerol, 2% (v/v) 2-mercaptoethanol, and 0.01% bromophenol blue and was boiled at 100°C for 5 min before electrophoresis. The extracted protein samples with equal amount (30 μg/lane) were loaded onto 10% sodium dodecyl sulfate polyacrylamide gel and transferred to a polyvinylidene difluoride membrane (Millipore, Billerica, MA, United States). Membrane blocking was conducted with 5% bovine serum albumin for 1 h and was incubated with primary antibodies (1:1,000) overnight at 4°C with gentle shaking. The membranes were washed three times with TBST (1× Tris buffered saline including 0.1% Tween 20) and then were incubated with a second antibody of horseradish peroxidase-conjugated (1:3,000, Cell Signaling Technology Inc., Beverly, MA, United States) at room temperature. The primary antibodies for phorsphor-LKB1 (Thr189, no. 3054s, 1:1,000), phorsphor-AMKPα (Thr172, no. 2531s, 1:1,000), and α-Tubulin (no. 2125s, 1:1,000) were purchased from Cell Signaling Technology Inc. (Beverly, MA, United States) and were validated previously for use with chicken samples (Zhang et al., 2017). The membranes were developed using ECL chemiluminescent reagents (Tanon Science and Technology Co., Ltd., Shanghai, China) and exposed to Kodak film. Band densities were quantified using Scion Image software (Scion Corporation, Frederick, MD, United States) and were normalized to α-tubulin and expressed as the relative values to those for the control group.

### Statistical Analysis

All data were statistically analyzed with the SAS program (version 8.02, SAS Institute Inc., Cary, NC, United States). All data were normally distributed and were analyzed using analysis of one-way analysis of variance. Significant differences among treatment means were analyzed by Duncan‘s multiple-range test. The results were presented with mean values with their standard deviation, and a *P* value of less than 0.05 was considered statistically significant.

## Results

### Growth Performance

The results of dietary supplemented with GAA on growth performance of Qiangdongnan Xiaoxiang chickens are shown in Table 3. Both 600 mg/kg GAA and 1,200 mg/kg GAA supplementation in diets did not affect ADG, ADFI, or F/G when compared with those fed with the control diet (*P* > 0.05). Similarly, there were no differences in ADG, ADFI, or F/G between 600 mg/kg GAA and 1,200 mg/kg GAA supplementation group (*P* > 0.05).

**TABLE 3:** Effect of dietary supplemented with GAA on growth performance of Qiandongnan Xiaoxiang Chicken.

**Items**	**Control group**	**600 mg/kg GAA group**	**1,200 mg/kg GAA group**	***P* value**
ADG (g)	24.60 ± 1.34	25.09 ± 3.44	23.86 ± 3.77	0.850
ADFI(g)	110.0 ± 4.43	105.8 ± 3.98	108.1 ± 4.80	0.426
F/G(g/g)	4.49 ± 0.36	4.27 ± 0.57	4.65 ± 1.02	0.754

*Results are expressed as the mean value and standard error. Means within the same row with different superscripts differ significantly (*n* = 40 per treatment, *P* < 0.05). Control, broilers fed the basal diet without transport stress; T 3h, broilers fed the basal diet and experienced a 3 h transport; T 3h + 0.06% GAA or T 3h + 0.12% GAA, broilers fed the basal diet supplemented with GAA at 600 or 1200 mg/kg and experienced a 3 h transport. GAA, guanidine acetic acid; ADG, average daily weight gain; ADFI, average daily feed intake; F/G, the ratio of feed to gain.*

### Plasma Parameters

**FIGURE 1:**
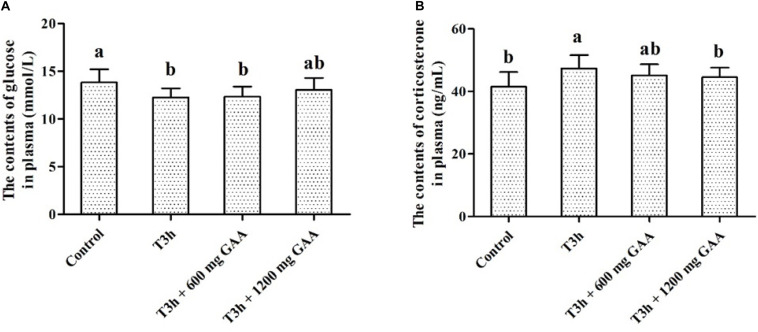
Effects of dietary GAA supplementation on the contents of glycose **(A)** and corticosterone **(B)** plasma of Qiandongnan Xiaoxiang Chicken experienced transport stress (*n* = 10 per treatment). Results are expressed as the mean value and standard error. Means within the same row with different superscripts differ significantly (*n* = 10 per treatment, *P* < 0.05). Control, broilers fed the basal diet without transport stress; T3h, broilers fed the basal diet and experienced a 3 h transport; T 3h + 0.06% GAA or T 3h + 0.12% GAA, broilers fed the basal diet supplemented with GAA at 600 or 1,200 mg/kg and experienced a 3 h transport. GAA, guanidine acetic acid.

The concentrations of glucose and corticosterone in plasma are shown in Figure 1. Compared with those in the control group (no pre-slaughter transport stress), the 3-h pre-slaughter transport significantly decreased the concentration of glucose and elevated the contents of corticosterone in plasma (*P* < 0.05, Figures 1A,B). However, dietary supplemented with 1,200 mg/kg GAA decreased the concentration of corticosterone of broilers induced by the 3-h pre-slaughter transport (*P* < 0.05). Moreover, there was no difference in the concentration of glucose or corticosterone between the control group and the 1,200-mg/kg GAA supplementation group (*P* > 0.05).

### Meat Quality

As shown in Table 4, compared with broilers without transport stress, pH_24__h_ values of broilers subjected to 3-h transport stress were significantly decreased (*P* < 0.05). In contrast, the drip loss, cooking loss, and L^∗^ value were all increased by 3-h transport stress (*P* < 0.05). However, the values of pH_45__min_, a^∗^, b^∗^, and shear force were not affected (*P* > 0.05). Diets supplemented with 600 mg/kg GAA or 1,200 mg/kg GAA similarly resulted in a higher value of pH_24__h_ and the lower drip loss and L^∗^ of broilers in comparison with those subjected to 3-h transport stress (*P* < 0.05). Besides, compared with 3-h transport stress, the cooking loss was lowered by 1200 mg/kg GAA supplementation (*P* < 0.05). However, both 600 mg/kg GAA and 1,200 mg/kg GAA supplemented did not affect the values of pH_45__min_, a^∗^, and b^∗^ (*P* > 0.05). Moreover, the drip loss of broilers fed with 1,200 mg/kg GAA was significantly lower than those fed with 600 mg/kg GAA diet (*P* < 0.05). No differences in parameters of meat quality measured in our study were observed between the 1,200-mg/kg GAA supplementation group and the control group (*P* > 0.05).

**TABLE 4:** Effects of dietary supplementation with GAA on meat quality of the pectoralis major muscle of Qiandongnan Xiaoxiang Chicken subjected to preslaughter transport stress.

**Items**	**Control group**	**T 3h**	**T 3h + 600 mg GAA**	**T 3 h + 1200 mg GAA**	***P* value**
pH_45__min_	6.40 ± 0.30	6.29 ± 0.22	6.34 ± 0.13	6.37 ± 0.07	0.720
pH_24__h_	5.88 ± 0.06^a^	5.59 ± 0.05^b^	5.86 ± 0.06^a^	5.87 ± 0.09^a^	< 0.001
L*	51.5 ± 1.15^c^	55.2 ± 1.61^a^	53.4 ± 1.69^b^	52.5 ± 1.60^bc^	< 0.001
a*	1.29 ± 0.11	1.21 ± 0.09	1.22 ± 0.18	1.25 ± 0.13	0.584
b*	5.08 ± 0.68	5.45 ± 0.28	5.06 ± 0.21	5.09 ± 0.33	0.220
Drip loss (%)	2.26 ± 0.21^c^	2.75 ± 0.18^a^	2.54 ± 0.12^b^	2.30 ± 0.24^c^	< 0.001
Cooking loss (%)	16.6 ± 0.73^b^	18.0 ± 1.18^a^	17.2 ± 0.14^ab^	16.8 ± 0.89^b^	0.011
Shear force (N)	24.1 ± 0.86	25.1 ± 1.22	25.1 ± 0.92	24.3 ± 1.11	0.141

Results are expressed as the mean value and standard error. Means within the same row with different superscripts differ significantly (*n* = 10 per treatment, *P* < 0.05). Control, broilers fed the basal diet without transport stress; T 3h, broilers fed the basal diet and experienced a 3 h transport; T 3h + 0.06% GAA or T 3h + 0.12% GAA, broilers fed the basal diet supplemented with GAA at 600 or 1,200 mg/kg and experienced a 3 h transport. GAA, guanidine acetic acid; pH_45__*min*_, pH at 45 min postmortem; pH_24__*h*_, pH at 24 h postmortem; L*, lightness; a*, redness; b*, yellowness.

### Concentrations of Cr, PCr, ATP, ADP, and AMP in Muscle

As shown in Table 5, compared with the control group, 3-h transport stress significantly decreased the contents of Cr and ATP in PM (*P* < 0.05) but elevated the contents of ADP, AMP, and the ratio of AMP: ATP (*P* < 0.05). However, no differences in the ratio of PCr:Cr and the concentration of PCr between 3-h transport stress and the control group were observed (*P* > 0.05). Compared with the 3-h pre-slaughter transport treatment, dietary supplemented with 600 mg GAA significantly increased the contents of Cr and PCr in PM (*P* < 0.05). However, the contents of ATP, ADP, and AMP and the ratio of PCr:Cr and AMP:ATP (*P* > 0.05) were not affected by dietary supplemented with 600 mg GAA when compared with 3-h transport stress. Notably, compared with 3-h pre-transport stress, dietary supplemented with 1,200 mg GAA exhibited significant increases in the contents of Cr, PCr, and ATP in muscle (*P* < 0.05). Furthermore, the contents of ADP and AMP and the ratio of AMP:ATP (*P* < 0.05) by 1,200 mg GAA supplementation were lower than those of 3-h transport stress. However, there were no differences in the ratio of PCr:Cr among four treatments (*P* > 0.05).

**TABLE 5:** Effects of dietary supplementation with GAA on muscle energy status of the pectoralis major muscle of Qiandongnan Xiaoxiang Chicken subjected to preslaughter transport stress.

**Items**	**Control group**	**T 3 h**	**T 3 h + 600 mg GAA**	**T 3 h + 1,200 mg GAA**	***P* value**
Cr	21.99 ± 1.36^b^	19.79 ± 1.46^c^	22.90 ± 1.60^ab^	24.58 ± 1.70^a^	0.001
PCr	2.24 ± 0.15^ab^	2.10 ± 0.17^b^	2.32 ± 0.16^a^	2.41 ± 1.48^a^	0.042
PCr: Cr	0.10 ± 0.01	0.11 ± 0.01	0.10 ± 0.01	0.10 ± 0.01	0.731
ATP	3.48 ± 0.16^a^	3.03 ± 0.13^c^	3.14 ± 0.20^bc^	3.35 ± 0.17^ab^	0.002
ADP	0.90 ± 0.03^b^	1.04 ± 0.09^a^	0.97 ± 0.06^ab^	0.94 ± 0.01^b^	0.009
AMP	0.32 ± 0.03^c^	0.42 ± 0.03^a^	0.38 ± 0.02^ab^	0.35 ± 0.02^bc^	0.001
AMP: ATP	0.09 ± 0.01^b^	0.14 ± 0.02^a^	0.12 ± 0.01^a^	0.10 ± 0.01^b^	< 0.001

*Results are expressed as the mean value and standard error. Means within the same row with different superscripts differ significantly (*n* = 10 per treatment, *P* < 0.05). Control, broilers fed the basal diet without transport stress; T3h, broilers fed the basal diet and experienced a 3 h transport; T3h + 0.06% GAA or T3h + 0.12% GAA, broilers fed the basal diet supplemented with GAA at 600 or 1,200 mg/kg and experienced a 3 h transport. GAA, guanidine acetic acid; Cr, creatine; PCr, phosphocreatine; ATP, adenosine triphosphate; ADP, adenosine diphosphate; AMP, adenosine monophosphate.*

### Glycolytic Potential of Pectoralis Major Muscle

**FIGURE 2:**
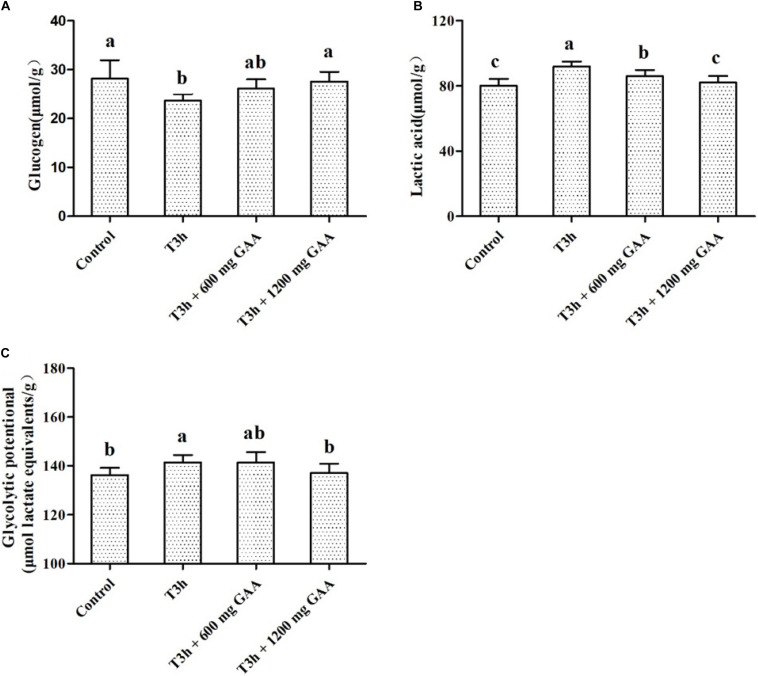
Effects of dietary GAA supplementation on concentrations of glycogen **(A)**, lactic acid **(B)**, and glycolytic potential **(C)** in pectoralis major muscle of Qiandongnan Xiaoxiang Chicken experienced transport stress (*n* = 10 per treatment). Results are expressed as the mean value and standard error. Means within the same row with different superscripts differ significantly (*n* = 10 per treatment, *P* < 0.05). Control, broilers fed the basal diet without transport stress; T3h, broilers fed the basal diet and experienced a 3 h transport; T3h + 0.06% GAA or T3h + 0.12% GAA, broilers fed the basal diet supplemented with GAA at 600 or 1,200 mg/kg and experienced a 3 h transport. GAA, guanidine acetic acid; glycolytic potential = 2 × [glycogen] + [lactic acid].

The results of the glycolytic potential of PM muscle of Qiandongnan Xiaoxiang chickens fed with GAA are shown in Figure 2. Three-hour pre-slaughter transport stress resulted in lower glycogen contents and higher lactic acid (*P* < 0.05, Figures 2A,B), combined with higher glycolytic potential of PM muscle (*P* < 0.05, Figure 2C) in comparison with those of the control group. The content of lactic acid was decreased followed by 600 mg/kg GAA supplementation (*P* < 0.05); however, no differences were observed for glycogen content or glycolytic potential by 600 mg/kg GAA supplementation (*P* > 0.05). Compared with the 3-h pre-slaughter transport, however, 1,200 mg/kg GAA addition significantly elevated the glycogen content (*P* < 0.05) and markedly decreased glycolytic potential and the concentration of lactic acid (*P* < 0.05). Moreover, the content of lactic acid of broilers supplemented with 1,200 mg/kg GAA was lower than those fed with 600 mg/kg GAA (*P* < 0.05), but there were no differences in glycogen content or glycolytic potential between these two groups (*P* > 0.05). Additionally, no differences were observed for glycogen, lactic acid, or glycolytic potential between the 1,200-mg/kg GAA group and the control group (*P* > 0.05).

### Activities of Glycolytic Key Enzymes

**FIGURE 3:**
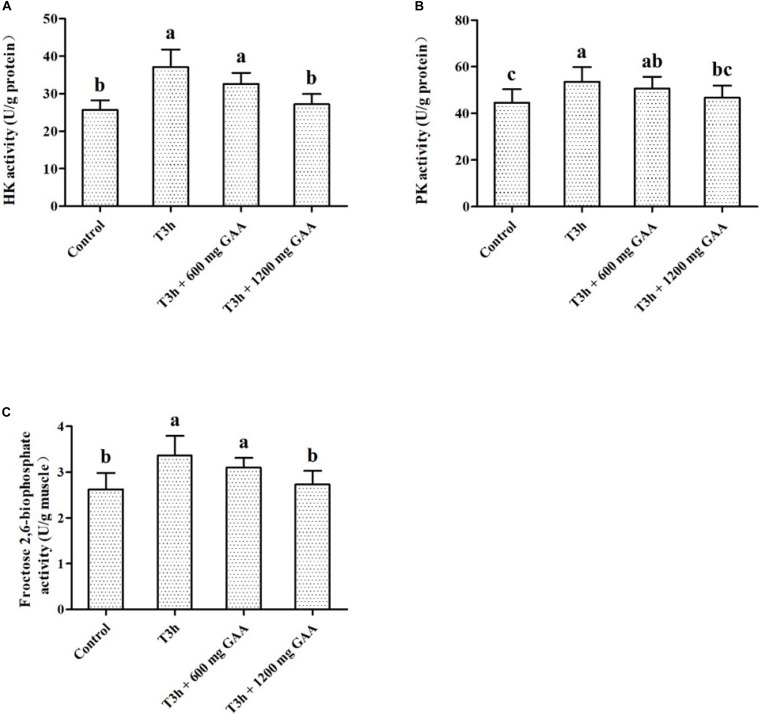
Effects of dietary GAA supplementation on activities of HK **(A)**, PK **(B)**, and Fructose 2,6-niophosphate **(C)** in pectoralis major muscle of Qiandongnan Xiaoxiang Chicken experienced transport stress (*n* = 10 per treatment). Results are expressed as the mean value and standard error. Means within the same row with different superscripts differ significantly (*n* = 10 per treatment, *P* < 0.05). Control, broilers fed the basal diet without transport stress; T3h, broilers fed the basal diet and experienced a 3 h transport; T3h + 0.06% GAA or T3h + 0.12% GAA, broilers fed the basal diet supplemented with GAA at 600 or 1,200 mg/kg and experienced a 3 h transport. GAA, guanidine acetic acid; HK, hexokinase; PK, pyruvate kinase.

Data on the effects of transport stress on activities of glycolytic key enzymes are shown in Figure 3. Three-hour pre-slaughter transport stress increased the activities of PK and HK (Figures 3A,B), as well as the activity of 2,6-PFK (*P* < 0.05, Figure 3C). The activities of PK, HK, and 2,6-PFK were not significantly influenced by the 600-mg/kg GAA treatment compared to those treated with 3-h transport stress (*P* > 0.05). In contrast, compared with the 3-h transport stress group, the activities of PK, HK, and 2,6-PFK were all dramatically decreased by 1,200 mg/kg GAA supplementation (*P* < 0.05). Furthermore, compared to the control group, there were no differences in the activities of PK, HK, or 2,6-PFK of broilers supplemented with 1,200 mg/kg GAA (*P* > 0.05).

### Related mRNA Expressions and Protein Abundances in the AMPK Signaling Pathway

**FIGURE 4:**
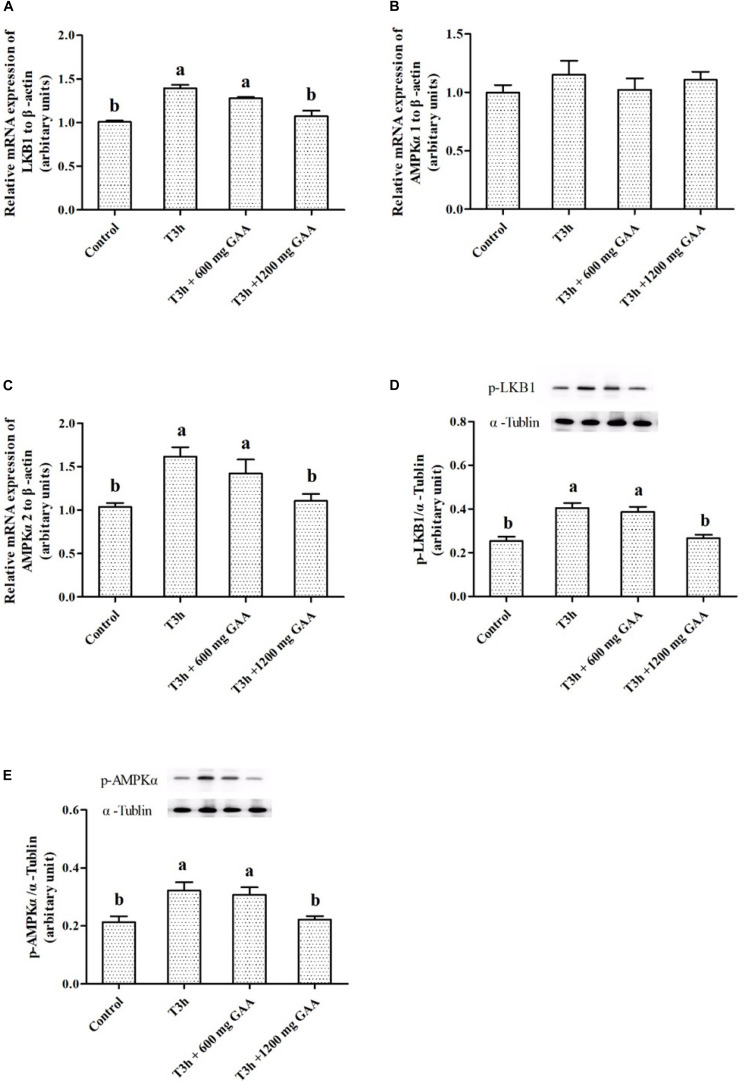
Effects of dietary GAA supplementation on mRNA expressions of LKB1 **(A)**, AMPKα1 **(B)**, AMPKα2 **(C)**, and protein abundances of phosphorylation LKB1 **(D)** and phosphorylation AMPKα **(E)** in pectoralis major muscle of Qiandongnan Xiaoxiang Chicken experienced transport stress (*n* = 10 per treatment). Results are expressed as the mean value and standard error. Means within the same row with different superscripts differ significantly (*n* = 10 per treatment, *P* < 0.05). Control, broilers fed the basal diet without transport stress; T3h, broilers fed the basal diet and experienced a 3 h transport; T3h + 0.06% GAA or T3h + 0.12% GAA, broilers fed the basal diet supplemented with GAA at 600 or 1,200 mg/kg and experienced a 3 h transport. GAA, guanidine acetic acid; LKB1, liver kinase B1; AMPKα1, adenosine 5′-monophosphate-activated protein kinase α1; AMPKα2, adenosine 5′-monophosphate-activated protein kinase α2; phosphorylation LKB1, phospho-liver kinase B1; phosphorylation AMPKα, phospho-adenosine 5′-monophosphate-activated protein kinase α (including α-1 and -2).

The mRNA expressions and related protein abundances are shown in Figure 4. Three-hour pre-slaughter transport stress resulted in a significant elevation of mRNA expressions of LKB1 and AMPKα2 (*P* < 0.05, Figures 4A,B), except for the mRNA expression of AMPKα1 (*P* > 0.05, Figure 4B). Meanwhile, protein abundances of LKB1 phosphorylation and AMPKα phosphorylation were both increased by 3-h transport stress (*P* < 0.05, Figures 4D,E). On the contrary, compared to those treated by 3-h transport stress, the upregulated mRNA expressions of LKB1 and AMPKα2, combined with the increased protein abundances of LKB1 and AMPKα, were all reversed by 1,200 mg/kg GAA supplementation (*P* < 0.05).

## Discussion

Several published studies have indicated that GAA supplementation could effectively increase creatine storage in tissues and accordingly positively affected the growth performance, breast meat yield, and status of energy metabolism when GAA was supplemented with a dosage not higher than 600–800 mg GAA per kg feed for chicken (Michiels et al., 2012; Mousavi et al., 2013; Ostojic, 2015). Surprisingly, in our study, there were no differences in ADG, ADFI, or F/G of broilers supplemented with 600 or 1,200 mg/kg GAA, which was probably associated with the shorter experimental period (14 days before pre-slaughter transport). Similarly, Zhang et al. (2019) suggested that dietary supplemented with GAA prior to pre-slaughter for 14 days at a dosage of both 600 and 1,200 mg/kg did not affect ADG, ADFI, or feed efficiency of broilers. Moreover, a previous study conducted by Tossenberger et al. (2016) also demonstrated that 0.6% GAA supplementation did not affect the growth performance of broilers.

The plasma parameters such as glucose and corticosterone were considered to be a biochemical index in response to pre-slaughter transport stress (Zhang et al., 2009; Voslarova et al., 2011). In our present study, 3-h pre-slaughter transport stress decreased the concentration of glucose in plasma, accompanied by an increase of corticosterone contents when compared with the control group, indicating that a stress occurred in response to 3-h pre-slaughter transport stress. Consistent with our results, Zhang et al. (2019) suggested that 3-h transport stress exhibited higher plasma corticosterone concentration and lower plasma glucose concentration than the control group (0.5-h transport stress). However, compared with those exposed to 3-h pre-slaughter transport, dietary supplemented with 1,200 mg/kg GAA resulted in a decrease in the concentration of corticosterone, demonstrating that GAA addition could contribute to alleviating the negative effects induced by the 3-h pre-slaughter transport.

Pre-slaughter transport causes an acute response to broilers, resulting in an inferior meat. In our present study, 3-h transport stress decreased pH_24__h_ values of broilers when compared with those in the control group. It was reported that the energy was generated from anaerobic glycolysis to maintain the metabolic activity of muscle cells during transport stress, resulting in the lactate and H^+^ accumulation, then followed by a lower pH (Xing et al., 2016). Moreover, the accumulation of lactate and protons from rapid anaerobic glycolysis induced by transport stress cannot be timely removed by postmortem meat, which may explain why pH at 24 h postmortem in the breast of 3-h transport broilers were still lower (Wang et al., 2017). In contrast, the drip loss, cooking loss, and L^∗^ value were all increased by 3-h transport stress. Similarly, Xing et al. (2016) demonstrated that the meat quality of broilers subjected to transport stress had higher drip loss, cooking loss, and L^∗^ value. Besides, Wang et al. (2017) also suggested that 3-h transport stress increased the L^∗^ value and drip loss of breast muscle at 24 h postmortem. Interestingly, in our present study, the broilers fed with both 600 mg/kg GAA and 1,200 mg/kg GAA for two weeks before slaughtering had a lower drip loss and L^∗^ value, combined with a higher value of pH_24__h_ in comparison with those subjected to 3-h transport stress. It was reported that GAA supplementation before slaughtering could enhance the contents of available energy sources such as PCr and ATP, which contributes to delaying the conversion of glycogen to lactic acid and consequently maintains a pH value postmortem. Accordingly, the higher pH value reduced muscle protein denaturation and increased muscle water holding capacity and meat tenderness, thus improving meat quality (Huff-Lonergan and Lonergan, 2005). Consistent with our results, Zhang et al. (2019) suggested that dietary addition of 1,200 mg/kg GAA reduced drip loss compared to 3-h pre-slaughter transport stress, which indicated that GAA supplementation prior to slaughtering could be an effective way to improve the meat quality of broilers subjected to transport stress.

It has been demonstrated that a lower ATP content and/or a higher AMP/ATP ratio in muscle were observed in broilers at the stage of pre-slaughter transport or heat stress (McKee and Sams, 1997; Savenije et al., 2002; Zhang et al., 2017). Similarly, our current study observed that 3-h transport stress decreased the contents of ATP, Cr, and PCr in muscle and increased AMP and the ratio of AMP:ATP, indicating that 3-h transport stress accelerated muscle ATP exhaust accompanied by the activation of the Cr and phosphocreatine system. The primary physiological function of Cr is involved in the regulation of energy metabolism of cells, particularly muscle cells, through the Cr and phosphocreatine system (Michiels et al., 2012). The Cr and phosphocreatine system is described as a spatial energy buffer because it acts as an energy transport system that carries high-energy phosphates from mitochondrial production sites to energy utilization sites (Brosnan and Brosnan, 2007). In addition, it is a temporal energy buffer because it maintains energy homeostasis by buffering ADP and ATP ratios in order to store and mobilize energy when required on short notice, especially in muscle cells (Lemme et al., 2007). It has been demonstrated that supplemental GAA, a natural precursor of Cr, serves as an efficient Cr source improving the muscle energy metabolism (Lemme et al., 2007). In our study, dietary supplementation GAA at a dosage of 600 or 1,200 mg/kg GAA increased the concentration of Cr and PCr in muscle when compared with the 3-h transport group. Similar with this, Michiels et al. (2012) demonstrated that GAA supplementation enhanced the contents of Cr and PCr in muscle, indicating that GAA was an efficient Cr source to improve energy store in the forms of Cr and PCr.

It has been demonstrated that pre-slaughter stress exacerbated skeletal muscle energy consumption, evidenced by decreasing muscle ATP depletion and accelerating glycolysis metabolism, which resulted in a decreased concentration of glycogen and an elevation of lactic acid (Yue et al., 2010; Zhang et al., 2017). Similar with the results of Zhang et al. (2019), in our present study, it showed that pre-slaughter transport stress increased the concentration of lactic acid and glycolytic potential of muscle, indicating that muscle cells switch from oxidative phosphorylation to glycolysis to produce enough ATP for increased muscle energy demands in response to the limited oxygen during pre-slaughter stress and anaerobic glycolysis then became the predominant energy source for the muscle ATP supply. It has been shown that HK, PK, and LDH are the rate-limiting enzymes involved in the glycolysis pathway, which are responsible for converting glucose to glucose-6-phosphate, phosphoenolpyruvate to pyruvic acid, and pyruvic acid to lactic acid, respectively (Liu et al., 2015; Wang et al., 2017). In the present study, 3-h pre-slaughter transport stress elevated the activities of PK, HK, and LDH of breast, indicating that glycolysis may be triggered during 3-h pre-slaughter transport stress. However, 1,200 mg/kg GAA supplementation increased the concentration of glycogen and decreased lactic acid, glycolytic potential and the activities of PK, HK, and LDH of breast compared to 3-h transport stress, indicating that the rate of glycolysis reaction was downregulated because of GAA supplementation. Moreover, compared to 3-h transport stress, the increased Cr and PCr in muscle were observed by GAA supplementation, indicating that GAA supplementation may be beneficial for delaying rapid muscle anaerobic glycolysis induced by transport stress.

AMPK, which is mainly recognized as an important regulator of mitochondrial biogenesis in response to energy deprivation, is switched on by an increase in the AMP/ATP ratio via the phosphorylation of the α subunit at the Thr^172^ site by LKB1 (Du et al., 2005). AMPK is activated by stresses that deplete cellular ATP, when it acts to restore energy homeostasis by switching on catabolic pathways that generate ATP through accelerating the glycolysis process of muscle (Graeme and Hardie, 2014). However, a previous study in AMPK knockout mice demonstrated that postmortem glycolysis was inhibited (Shen et al., 2008), which suggested that AMPK played an important role in the postmortem glycolysis process in lack of oxygen. Moreover, AMPK is a heterotrimeric complex comprising α, β, and γ subunits, in which the α subunit is essential for the activation of AMPK signaling (Du et al., 2005; Graeme and Hardie, 2014). The α subunit of AMPK has two isoforms, α1 and α2. It was demonstrated that AMPKα2 but not AMPKα1 knockoff abolished the activity of AMPK in postmortem muscle. Besides, AMPKα2 knockoff reduced postmortem pH decline and the generation of lactate, while AMPKα1 knockoff had no significant effect, which suggested that the AMPKα2 catalytic subunit mainly regulates postmortem glycolysis in muscle (Liang et al., 2013). In our present study, it was observed that both LKB1 mRNA expression level and LKB1 protein abundances, combined with the phosphorylation abundance of AMPKα at Thr^172^, were all increased by 3-h transport stress, indicating that the energy metabolism of muscle under pre-slaughter transport stress was mediated by AMPK signaling. Similarly, it has been suggested that pre-slaughter transport stress accelerated the process of glycolysis, recognized by increasing an accumulation of lactate and subsequently an activation of the AMPK signaling pathway (Xing et al., 2016). Meanwhile, our study also indicated that AMPKα2 but not AMPKα1 was elevated in response to 3-h transport stress. Nevertheless, dietary GAA supplementation downregulated the mRNA expressions of muscle LKB1 and AMPKα2, and the protein expression of LKB1 and the phosphorylation abundance of AMPKα Thr^172^, suggesting that dietary GAA addition may be an effective way in delaying anaerobic glycolysis via inhibiting the transport stress-induced activation of the AMPK pathway.

## Data Availability Statement

The original contributions presented in the study are included in the article/supplementary material, further inquiries can be directed to the corresponding author/s.

## Ethics Statement

The animal study was reviewed and approved by Institutional Animal Care and Use Committee of Zunyi Normal College.

## Author Contributions

RL, JL, and NL participated in the animal trial and sample analysis together with BZ. BZ performed the data analysis and wrote the manuscript. ZH, PS, MH, and YX assisted in conducting the experimental analysis. BZ and ZS designed and supervised the study and revised the manuscript. All authors contributed to the article and approved the submitted version.

## Conflict of Interest

The authors declare that the research was conducted in the absence of any commercial or financial relationships that could be construed as a potential conflict of interest.
